# Long-Term Follow-Up of a Randomized Controlled Trial to Reduce Excessive Weight Gain in Infancy: Protocol for the Prevention of Overweight in Infancy (POI) Follow-Up Study at 11 Years

**DOI:** 10.2196/24968

**Published:** 2020-11-30

**Authors:** Taiwo O Adebowale, Barry J Taylor, Andrew R Gray, Barbara C Galland, Anne-Louise M Heath, Sarah Fortune, Kim A Meredith-Jones, Trudy Sullivan, Deborah McIntosh, Bradley Brosnan, Rachael W Taylor

**Affiliations:** 1 Department of Medicine University of Otago Dunedin New Zealand; 2 Department of Women's and Children's Health Children's Pavilion Dunedin Public Hospital University of Otago Dunedin New Zealand; 3 Biostatistics Centre University of Otago Dunedin New Zealand; 4 Department of Women's and Children's Health University of Otago Dunedin New Zealand; 5 Department of Human Nutrition University of Otago Dunedin New Zealand; 6 Department of Psychological Medicine University of Otago Dunedin New Zealand; 7 Department of Preventive and Social Medicine University of Otago Dunedin New Zealand

**Keywords:** infant, child, obesity, prevention, sleep, physical activity, mental wellbeing, screen time, diet

## Abstract

**Background:**

The Prevention of Overweight in Infancy (POI) randomized controlled trial assessed the effect of a more conventional food, physical activity, and breastfeeding intervention, with a more novel sleep intervention on weight outcomes at 2 years of age. The trial had 58% uptake at recruitment, and retention was 86% at age 2 years, 77% at age 3.5 years, and 69% at age 5 years. Children who received the brief sleep intervention in infancy had just half the risk of obesity at 2 years of age compared to those who did not receive the sleep intervention. Importantly, this substantially reduced risk was still apparent at our follow-up at 5 years of age.

**Objective:**

The primary aim of this follow-up at age 11 years is to determine whether differences in BMI z-score and obesity risk remain apparent now that it is at least 9 years since cessation of the sleep intervention. Several secondary outcomes of interest will also be examined including 24-hour movement patterns, mental health and wellbeing, and use of electronic media, particularly prior to sleep.

**Methods:**

We will seek renewed consent from all 734 of the original 802 POI families who expressed interest in further involvement. Children and parent(s) will attend 2 clinics and 1 home appointment to obtain measures of anthropometry and body composition (dual-energy x-ray absorptiometry scan), 24-hour movement patterns (sleep, sedentary time, and physical activity measured using an AX3 accelerometer), mental health and wellbeing (validated questionnaires), family functioning (validated questionnaires), use of electronic media (wearable and stationary cameras, questionnaires), and diet and eating behaviors (24-hour recall, questionnaires).

**Results:**

This follow-up study has full ethical approval from the University of Otago Human Ethics Committee (H19/109) and was funded in May 2019 by the Health Research Council of New Zealand (grant 19/346). Data collection commenced in June 2020, and first results are expected to be submitted for publication in 2022.

**Conclusions:**

Long-term outcomes of early obesity intervention are rare. Despite the growing body of evidence linking insufficient sleep with an increased risk of obesity in children, interventions targeting improvements in sleep have been insufficiently explored. Our initial follow-up at 5 years of age suggested that an early sleep intervention may have long-term benefits for effective weight management in children. Further analysis in our now preteen population will provide much-needed evidence regarding the long-term effectiveness of sleep interventions in infancy as an obesity prevention approach.

**Trial Registration:**

ClinicalTrials.gov NCT00892983; https://tinyurl.com/y3xepvxf

**International Registered Report Identifier (IRRID):**

DERR1-10.2196/24968

## Introduction

Childhood obesity remains a serious global health challenge [1], with an estimated 38.2 million children under the age of 5 years [2] and over 340 million children and adolescents aged 5-19 years considered overweight or obese [1]. New Zealand is no exception; more than 33% of those aged 2-14 years are overweight or obese [3], with Māori (the indigenous population of New Zealand) and Pasifika children and those living in areas of greater socioeconomic need being disproportionately affected [3-5]. Obesity in childhood persists into adulthood, with risks to immediate and future health and wellbeing [6-8]. The costs of childhood obesity, including drug therapy, are high and rising yearly [9-11]. Drug therapy for obesity at any time, either in childhood or adulthood, is fraught with difficulties and often results in failure [12,13]. While genetic factors play a role in overweight and obesity, they are unlikely to explain the rapid rise in global obesity rates [14-17]. Unhealthy lifestyles, within the larger context of environmental, social, and economic forces, play a crucial role in the childhood obesity epidemic [17,18].

Despite evidence suggesting that the foundations for good health are laid down in the early years of life [19-21], until recently, relatively little attention has been paid to investigating the potential for obesity prevention in infants and toddlers [22-24]. To date, preventive approaches have largely focused on dietary intake [25-27] and physical activity [25-28]. For all their promise [29,30], initial findings from studies targeting nutrition, eating behaviors, physical activity, or sedentary behavior have been somewhat disappointing, perhaps due in part to many being underpowered or short-term [22,24,26,31-33]. However, a growing number of large randomized controlled trials (RCTs) aimed at promoting healthy eating behaviors, improving physical activity, and reducing sedentary time in infants and toddlers have also had somewhat mixed success in reducing excessive weight gain during the early years [27,34-36]. The Healthy Beginnings trial, undertaken in socioeconomically disadvantaged areas of Sydney, Australia, reported important group differences in mean BMI at the end of the intervention at 2 years of age [35]. However, these benefits had disappeared when the children were followed up at 5 years of age [37]. The Insight study, based in the United States, reported a modest reduction in BMI *z*-scores in children at the end of the 3-year intervention [36], but planned follow-up data have not been published to date. Other interventions aiming to improve infant feeding and parental feeding behaviors or physical activity and sedentary behavior have not produced significant benefits in terms of BMI, although they do report some important and relevant effects on obesity-related behaviors [34,38]. It seems that the prevention of overweight and obesity in childhood through changing dietary intake or physical activity levels at a young age is more challenging than initially anticipated [29,39-41].

Thus, other approaches require consideration [31]. One behavior receiving considerable attention more recently is the importance of good sleep for effective weight management in children. Multiple meta-analyses of observational studies have demonstrated a strong and consistent association between shorter sleep duration and increased obesity risk in children, including in the early years [42-44]. However, very few intervention trials have determined the impact of influencing sleep behavior in the first 1000 days (conception to 2 years of age) on weight outcomes in young children. The Insight intervention focused on responsive feeding and healthy dietary choices from infancy through to 3 years of age but also included messages relating to sleep [36]. Overall, the intervention was effective at reducing mean BMI z-score, but as the study involved a multicomponent intervention, the relative contribution of the sleep intervention cannot be isolated.

In 2009, we commenced the Prevention of Overweight in Infancy (POI) study, a 4-group RCT in 802 families that aimed to reduce the number of children with excessive weight gain in the first 2 years of life [45]. Women in the latter stages of pregnancy were randomized and stratified by parity and socioeconomic status, with equal probabilities to receive the following from late pregnancy through infancy: (1) Control (usual care); (2) conventional food, physical activity, and breastfeeding intervention (FAB); (3) more novel Sleep intervention; (4) Combination group (received both FAB and Sleep interventions). All participants received standard Maternity and Well Childcare [45]. Further details are available in the original study protocol and outcome papers [45-47]. No significant effect on BMI z-score was observed between intervention and usual care groups at the end of the intervention when children were 2 years of age. However, an exploratory analysis showed that children who had received the sleep intervention (including those from both the Sleep and Combination groups) had half the odds of obesity as children who did not receive the sleep intervention [47]. Given this promising effect of sleep, a follow-up study was undertaken with the cohort at 3.5 years of age and 5 years of age (just prior to starting school). Interestingly, the benefit of the sleep intervention remained apparent at 5 years of age, despite no intervention having occurred for at least 3 years. Importantly, statistically significant differences were observed both in terms of mean BMI z-score (–0.23, 95% CI –0.38 to –0.07) and in the number of children classified as obese (relative risk 0.49, 95% CI 0.28 to 0.84) [46-48]. The reduction in both mean BMI *and* the number of children at the higher end of the BMI distribution as a result of our sleep intervention is a very important outcome from a public health perspective.

Our finding of a clinically important difference in obesity rates at 5 years of age following a brief intervention that finished many years previously clearly warrants further follow-up given the public health implications of this finding, particularly given the dearth of long-term follow-up of obesity prevention trials in children [49]. The POI children are now 11 years of age, providing an invaluable opportunity to assess the long-term effects of the initial intervention. Other variables of interest including sleep, dietary, and activity behaviors; body composition; physical and mental health and wellbeing; family functioning; and use of electronic media will also be examined, as outlined in our methods.

The primary aim of this follow-up study is to determine whether differences in BMI z-score and obesity risk remain apparent between those who did and those who did not receive early sleep intervention at least 9 years after completion of the intervention.

Secondary objectives relating to the RCT are to determine whether early sleep intervention has long-term effects on sleep, physical activity, and dietary intake; influences body composition in pre-adolescents; and impacts pre-adolescent physical and mental health and wellbeing.

Secondary objectives relating to cross-sectional and cohort analyses include associations between early 24-hour movement behaviors (sleep, sedentary time, physical activity) and subsequent body composition and bone health, whether 24-hour movement behaviors (sleep, sedentary time, physical activity) are associated with physical and mental health and wellbeing in children, and how preteens use electronic media before bed and associations with sleep and mental health and wellbeing.

## Methods

### Study Design and Participants

The original 4-arm POI RCT determined the impact of sleep and more conventional food and activity interventions (individually and in combination) on weight gain from birth until 2 years of age. Eligible participants were pregnant women aged 16 years or older; able to communicate in English or Te Reo Māori (the indigenous language of New Zealand); resided in Dunedin, with no plan of leaving the local area before the child’s second birthday; and were booked into the Queen Mary Maternity Unit, Dunedin Hospital at 28-30 weeks’ gestation, or their Lead Maternity Carer had notified the POI team of their expected home birth before 34-week gestation. Queen Mary is the main birthing unit where most births (≥97%) occur in Dunedin (the remaining 3% are home births). After birth, children born before 36.5 weeks’ gestation and children with any form of congenital abnormality that could potentially affect feeding and growth were excluded from the study. Based on the inclusion and exclusion criteria, a total of 802 pregnant women took part in the original RCT [46]. Retention rates were 86% at 2 years, 77% at 3.5 years, and 69% at 5 years of age [46,47].

All participants from the original RCT, except for 68 families who had requested no further contact, will be invited for this follow-up at 11 years of age. Full ethical approval for the 11-year-old measures has been obtained from the University of Otago Human Ethics Committee (H19/109), and written informed consent will be obtained from both child and parent participants before this follow-up study commences.

### Sample Size

Given the 734 families who have indicated an interest in future follow-up and the high retention rates at baseline and first follow-up measurement when the children turned 3.5 years old and 5 years old [47], we estimate that at least 500 (62%) of the original cohort will be retained in this follow-up. This number of participants will provide 80% power to detect differences in BMI z-scores of 0.23 between 2 approximately equally-sized groups of those who did and those who did not receive early sleep intervention (observed differences at 3.5 years of age and 5 years of age were 0.24 and 0.23, respectively) assuming an SD of 0.90 (estimated from data at 3.5 years of age and 5 years of age) using two-sided *P*<.05.

### Data Collection

All outcome data will be collected at 3 scheduled appointments: 2 visits to our University research clinic rooms (60-90 minutes each) and 1 (15-45 minutes) home visit. At the first visit, children will be weighed and measured and undergo a dual-energy x-ray absorptiometry (iDXA) scan to assess body composition. Both the child and their parent will complete questionnaires as detailed in the following sections, and children will be fitted with an accelerometer to be worn over the following week. At the second clinic visit, a 24-hour recall will be conducted, and additional questionnaires will be completed. Researchers will visit each home during the experimental week to set up the wearable and stationary cameras and to collect a second 24-hour recall in a random subsample of children. At the end of the study, children will be given a NZ $100 gift voucher for their participation.

### Outcome Measures

#### Overview

Outcome measures for the 11-year-old follow-up are presented in Table 1. The methods used for each measure are also highlighted [50-62]. All follow-up measurements will be carried out, as per standard protocols, by research staff who are trained and blinded to the original group allocation [50]. As our interest is in real-world effects from offering the intervention, the main analyses will be performed on a modified intention-to-treat basis (using all available data, with multiple imputation used for the primary outcome as described in later sections). The primary outcomes are BMI z-score and relative risk of obesity at 11 years of age [45,48]. Other measures described herein are related to the secondary objectives at the 11-year-old follow-up.

**Table 1:** Outcome measures at 11 years.

Category, method		Measure	Child (11 years old)	Parent
**Anthropometry**				
	International Growth Reference [50]	Height, weight, BMI-for-age z score	√	N/A^a^
	International Growth Reference [50]	BMI	√	N/A
	Calculated BMI z-score ≥85th but ≤95th percentile	Prevalence of overweight	√	N/A
	BMI z-score ≥95th percentile [50]	Prevalence of obesity	√	N/A
**Blood pressure (BP)**				
	Automated BP Monitor (Omron)	Systolic and diastolic BP	√	N/A
**Body composition and bone health**				
	DXA^b^ scan	Body fat (g)	√	N/A
	DXA scan	Lean mass (g)	√	N/A
	DXA scan	Bone mass (g)	√	N/A
	DXA scan	Bone mineral content (g/cm^2^)	√	N/A
**Mental health and wellbeing**				
	Strengths and Difficulties Questionnaire (SDQ) [51]	Emotional symptoms, conduct problems, hyperactivity/inattention, peer relationship problems, prosocial, behavior problems	√	√
	Child Health Utility 9D instrument [52,53]	Health-related quality of life	√	N/A
	The WHO^c^-5 Well-Being Index [54]	Current mental wellbeing	√	N/A
**Family functioning**				
	McMaster Family Assessment Device (FAD) 12-item General Functioning Scale [55]	General functioning	N/A	√
**Sleep**				
	AX3 accelerometer	Sleep timing, duration, and awakenings	√	N/A
	Adolescent Sleep Hygiene Scale [61]	Sleep hygiene	√	N/A
	PROMIS^d^ Pediatric Sleep Disturbance [62]	Sleep disturbances	√	√
	PROMIS Pediatric Sleep Impairment [62]	Sleep problems	√	√
**Physical activity and sedentary behavior**				
	AX3 accelerometer	Physical activity and sedentary behavior	√	N/A
**Dietary intake**				
	24-hour dietary recall [56]	Food and nutrient intake	√	N/A
	Camera (food images)	Food and nutrient intake	N/A	√
	Child Eating Behavior Questionnaire [57]	Eating behaviors	N/A	√
**Screen time**				
	Bedtime and electronic devices recall	Pre-bed and in-bed screen use	√	N/A
	Wearable and bedroom camera	Pre-bed and in-bed screen use	√	N/A
	Youth Activity Profile [58]	Total daily screen use	N/A	√
**Economic analyses**				
	BMI z-score at 2, 3.5, 5, and 11 years of age	Retrospective cost-effectiveness analysis	N/A	N/A
	BMI z-score at 2, 3.5, 5, and 11 years of age	Incremental cost-effectiveness ratios	N/A	N/A
	Child Health Utility 9D instrument [52,53]	Numbers and percentages	N/A	N/A

^a^N/A: not applicable.

^b^DXA: dual-energy x-ray absorptiometry.

^c^WHO: World Health Organization.

^d^PROMIS: Patient-Reported Outcomes Measurement Information System.

#### Anthropometry

All anthropometric measurements will be conducted following standard protocols by trained measurers [50]. Weight in kilograms will be obtained with children wearing light clothing using regularly calibrated electronic weight scales (Wedderburn WM206). Height (cm) will be measured using a wall-mounted Harpenden Stadiometer (Holtain Ltd, Crymych, United Kingdom). Both measurements will be obtained in duplicate, with a third measurement undertaken if duplicate measures are not within 0.1 kg for weight and 0.5 cm for height [45]. Where 3 measurements are recorded, the mean of the 2 closest values will be used; if all 3 measurements are equally spaced, the overall mean (or, equivalent, the median) will be used. BMI z-scores will be calculated using the World Health Organization growth reference for 5-19 years, with overweight defined as a BMI z-score ≥85th percentile but <95th percentile and obesity defined as ≥95th percentile [50].

#### Sleep, Physical Activity, and Sedentary Time

A single AX3 accelerometer (Axivity, Newcastle, UK) [59] will be used to measure 24-hour movement patterns (sleep, physical activity, sedentary time) [60] over 1 week. The accelerometer will be worn on the nondominant wrist (flexible wrist strap) 24 hours a day for 7 days including during showering, bathing, swimming, and other water-based activities. Children will complete the Adolescent Sleep Hygiene Scale [61] and the pediatric version of the 8-item Patient-Reported Outcomes Measurement Information System (PROMIS) Pediatric Sleep Disturbance and 8-item Sleep-Related Impairment questionnaires [62]. The Adolescent Sleep Hygiene Scale has good reliability with high internal consistency (Cronbach α of .84 in children of a similar age group as our study) [63]. Parents will also complete the proxy versions of the PROMIS questionnaires. These questionnaires provide a subjective assessment of difficulties with falling asleep and staying asleep, as well as daytime sleepiness in children, and the impact on functioning [62]. The PROMIS Pediatric Sleep Disturbance and Sleep-Related Impairment questionnaires have reliabilities of 0.91 and 0.88 respectively in children aged 8-17 years [64]. Children will self-complete 6 questions from the Youth Activity Profile [58], a self-report tool designed to assess physical activity and sedentary behavior in youth.

#### Dietary Intake and Eating Behavior

Dietary intake will be assessed using a multiple-pass 24-hour recall similar to the National Health and Nutrition Examination Survey multiple-pass method [56] but administered in person and supplemented with digital camera images of food taken before each eating occasion. The multiple-pass method entails a structured interview with the child to recall all food and drink consumed over the previous day (from midnight to midnight). A quick list of all foods and beverages consumed in the previous day is obtained (first pass). Additional information on the time and place of eating, along with camera images of food taken before meals, will be used to help aid recall (second pass). Comprehensive information regarding each food and drink item is collected including brand name; recipe, if known; and portion size (third pass). To assist this process, a variety of aids will be used to help participants report portion size including measuring cups, spoons, portion charts, and images of common food and beverage brands on an online shopping website from a major supermarket chain [65]. In addition, a list of typically forgotten foods will be presented. Finally, the 24-hour dietary recall will be reviewed with the child, and any additional food item(s) recalled will be added to the recall list (fourth pass). Recall data will be collected across all days of the week in the total sample, aiming for an approximately equal number of participants for each day of the week. A second recall will be collected on a different day of the week in a random sample of 50 participants in order to estimate “usual” intake using the Multiple Source Method, which enables estimates of usual dietary intake using data from multiple 24-hour recalls. The parent-report 35-item Child Eating Behavior Questionnaire [57] will be used as a subjective measure of eating style in children. The Child Eating Behavior Questionnaire has good internal consistency and reliability for food responsiveness (α=.83), emotional overeating (α=.70), and satiety responsiveness (α=.84). The questionnaire assesses eating style across 8 scales, namely food responsiveness, enjoyment of food, emotional overeating, desire to drink, satiety responsiveness, slowness in eating, emotional undereating, and food fussiness.

#### Body Composition, Bone Health, and Blood Pressure

Body composition and bone health will be assessed by DXA (Lunar iDXA; GE Healthcare, Madison, WI), performed and analyzed by one experienced operator with Lunar enCORE software version 18.0. This is the most advanced DXA system with a high degree of clarity, precision, and image resolution. The Lunar iDXA will determine the total body fat mass (kg) and the fat content of specific anatomical regions including trunk and extremity fat (these are automatic default regions) and central and peripheral fat (manual regions of interest) [66,67]. The percent coefficient of variation (%CV) for repeated in vivo scans on 10 adults from our laboratory is 1.3% for fat mass, 0.9% for lean mass, 1.8% for fat percentage, and 1% for total body bone mineral density. Blood pressure (BP) will be assessed using OMRON HEM-7211 upper arm BP monitors (Omron Healthcare Inc, Kyoto, Japan). These monitors are clinically proven to be accurate and validated within ±3 mm Hg for both systolic and diastolic BP readings in children (3-12 years old) and the adult population. Three readings will be obtained, with a fourth measurement taken if there is a difference of more than 10 mm Hg between the second and third systolic measurements. The mean of the second and third or 2 closest BP readings will be used. Following clinical practice guidelines for the screening of high BP in children and adolescents, readings higher than 100-119 mm Hg for systolic and 65-76 mm Hg for diastolic will be categorized as high [68,69].

#### Wellbeing and Family Functioning

Mental health, wellbeing, and family functioning will be assessed using several validated questionnaires. To assess wellbeing, both the child and parent will complete the 25-item Strength and Difficulties Questionnaire [51]. This is a standardized measure of overall emotional and behavioral wellbeing in children and young people with subscales of emotional symptoms, conduct problems, hyperactivity/inattention, peer relationship problems, and prosocial behavior [51]. The internal consistency and reliability of the Strength and Difficulties Questionnaire in adolescents aged 11-17 years and their parents is 0.70 [70]. Children will also complete the Child Health Utility 9D instrument [52,53], a pediatric generic preference-based measure of health-related quality of life, consisting of 9 dimensions with 5 levels in each [52,53]. In youth aged 8-12 years and 13-17 years, the Child Health Utility 9D instrument has a Cronbach α score of .77 and an intraclass correlation coefficient of .65 [71]. The World Health Organization WHO-5 Well-Being Index [53], a brief 5-item measure of current mental wellbeing for children aged 9 years and older, will also be completed by the child. Parents will complete the 12-item General Functioning Subscale of the McMaster Family Assessment Device [55] to assess family functioning. This is a validated single-index measure of the overall level of family functioning in areas of problem solving, behavior control, communication, roles, affective responsiveness, affective involvement, and general family functioning [55], giving insight into the relationships between parental efficacy, warmth, family rules, and the outcomes of interest (both obesity and mental health) [55]. The 12-item General Functioning Subscale has a good Cronbach α score of .86 with parents of children aged 4-16 years old.

#### Screen Time

Total daily screen use will be assessed using 6 questions from the Youth Activity Profile [58] completed by the children. We are also interested in the impact of electronic media before sleep — both in the hours before bed and once in bed. Due to the inherent difficulties in obtaining an accurate assessment of screen use in children, we will use an objective-driven approach to measure both pre-bed and in-bed screen use using wearable and stationary video cameras (PatrolEyes SC-DV7 Ultra 1296p Body Camera with Night Vision). One camera will be worn by the child via a chest harness (facing outwards so directly observes screen use) from 5 pm until bedtime in the home environment on a single night. The second infrared camera will be placed on a tripod in the room where the child sleeps and will capture screen use from 30 minutes before bedtime until the child gets out of bed in the morning. Objective data from the PatrolEyes camera with Night Vision will be used to measure screen device use throughout the night. Extensive guidelines are discussed with families to ensure that ethical obligations are met, including those that center around privacy issues [72]. We have also developed a new brief Bedtime and Electronic Devices recall to assess screen use both before bed and once in bed. Children will complete this recall about screen-based activities on the night before, on 2 days, including the day after the cameras. We will use data from a similar population of Dunedin children collected as part of a separate but associated study (Bedtime and Electronic Devices study, n=85 children aged 10.0-14.9 years with 4 days of camera data and 7 days of recall data) to determine whether reactivity to the camera (change in behavior from knowing the camera is present) has occurred.

#### Economic Analyses

Cost-effectiveness analysis will be conducted at the child level and based on intention-to-treat. The incremental cost per BMI z-score reduction for the Sleep intervention and the Combination (FAB and Sleep) groups will be compared to usual care (Control group). Costs will include the cost of delivering the Sleep and Combination interventions (eg, training of nurses, antenatal sessions, home visits, additional support, and written resources) and health care costs (eg, hospital, pharmaceutical, and general practitioner costs). Bootstrapping will be used to generate cost-effectiveness acceptability curves that estimate the probability the Sleep and Combination interventions are cost-effective for a range of willingness-to-pay thresholds. A health funder perspective will be reported for the analysis.

### Statistical Analyses

Based on results from earlier phases [46,47], the primary analysis will compare BMI z-scores between those receiving the Sleep intervention and those not (after checking for evidence of interaction between the Sleep and FAB interventions, these interactions were *P*≥.261 for 3.5 years and 5 years). In the absence of such interactions, as with ages 3.5 years and 5 years [47], multiple imputation using chained equations will be used and BMI z-score compared between the 2 groups using linear regression adjusting for stratification variables and the FAB intervention. Models will also examine secondary outcomes including overweight and obesity, estimating relative risks using Poisson regression with robust standard errors. Internal consistency will be assessed for all scales using Cronbach α with values ≥.80 considered to be at least good and values .70-.79 considered acceptable. For continuous outcomes where the assumptions required for linear regression cannot be satisfied, even after investigating natural logarithmic transformations, we will use quantile regression to model medians. If evidence of an interaction between the 2 interventions (Sleep and FAB) is present at this age, the interaction term will be retained, and otherwise identical 4-group comparisons will be presented instead. Standard model diagnostics will be used. Analyses will be conducted using Stata 16.1 and/or R 4.0.2 (or later versions), and 2-sided *P*<.05 will be considered statistically significant. As noted earlier, multiple imputation with chained equations will be used to accommodate missing-at-random data for BMI using the same auxiliary variables and parameters as previously [47]. While it is possible that informative missing data mechanisms exist, for example less enthusiasm to participate for children with very high BMIs, it would be surprising if such effects differed by their intervention group 9 years later. As was the case with our age 3.5 years and 5 years analyses [47], we will impute separately by intervention group and consider informative missingness mechanisms through exploring plausible scenarios adjusting imputed values in order to assess the robustness of our findings.

## Results

This follow-up study has full ethical approval from the University of Otago Human Ethics Committee (H19/109) and was funded in May 2019 by the Health Research Council of New Zealand (grant 19/346). Data collection commenced in June 2020, and first results are expected to be submitted for publication in 2022. Data collection will only take place while New Zealand is in Alert Levels 1 or 2 (physical distancing and appropriate hygiene recommendations) during the COVID-19 pandemic. The Otago region in New Zealand, where all data collection will take place, has been in Level 1 for all but 7 weeks of 2020 as overall case numbers in New Zealand remain extremely low (<2000 in a population of more than 5 million). As daily life is essentially normal in Level 1 with the exception of closed international borders, we feel confident that the pandemic will have relatively little effect on our data.

## Discussion

Although long-term follow-up of early life interventions for preventing childhood overweight and obesity is crucial in order to understand the health and economic benefits of differing approaches [49,73-75], few such analyses exist [49]. Most follow-ups to date are relatively short-term, all less than 5 years in duration (and many considerably less than this) [22,31,37], making it necessary to infer longer-term outcomes. Given the earlier promising results of our sleep intervention [46] and the paucity of obesity interventions reporting long-term outcomes [49], it is timely to undertake this follow-up, which is at least 9 years post-intervention. Our POI study also provides the opportunity to examine multiple influences on sleep and weight that are important during childhood, utilizing objective measures (7-day accelerometry and time-stamped cameras) to assess 24-hour movement patterns. Combined with the use of other novel measures to assess the use of electronic media prior to sleep, our analyses should also provide much-needed objective information about screen and sleep behaviors that can be difficult to measure accurately in children. Previous studies have shown that children’s sleep is particularly vulnerable to screen use [76,77], yet there is a dearth of objective data in this area (both screens and sleep), especially the critical information concerning pre-bedtime screen use. There is a clear need for a longer-term follow-up of early life obesity prevention approaches to inform effective risk reduction models [49,74,75]. This study aims to provide insight into the potential benefits of early sleep intervention for health and wellbeing in pre-adolescent children, providing a prevention strategy that could contribute to a new narrative about childhood obesity prevention.

## Appendix

Multimedia Appendix 1 Peer-review reports from Health Research Council New Zealand.

## Abbreviations

BP: blood pressure
DXA: dual-energy x-ray absorptiometry
FAB: food, physical activity, and breastfeeding
FAD: McMaster Family Assessment Device
POI: Prevention of Overweight in Infancy
PROMIS: Patient-Reported Outcomes Measurement Information System
RCT: randomized controlled trial
SDQ: Strength and Difficulties Questionnaire
WHO: World Health Organization
