# Supplementary material for: Long-Term Follow-Up of a Randomized Controlled Trial to Reduce Excessive Weight Gain in Infancy: Protocol for the Prevention of Overweight in Infancy (POI) Follow-Up Study at 11 Years
Source: JMIR Res Protoc. 2020 Nov 30;9(11):e24968. doi: 10.2196/24968 (PMC7735907; doi:10.2196/24968)
Supplement: Multimedia Appendix 1 [file resprot_v9i11e24968_app1.pdf]

Applicant peer review report

Health Research Council of New Zealand

Reviewer # 34

Proposal details

Title	Does a brief sleep intervention in infancy have long-term health benefits?
First named investigator	Professor Rachael Taylor (University of Otago)

Rationale for research — Score: 6

This application really has two parts. One is a follow-up of a strikingly successful sleep intervention targeting overweight and obesity. The effects have been very strong and long-lasting, and this is the 10-year follow up. This alone justifies the proposal, but there are a number of "add-ons". Some of these are directly related to the primary outcome: Are there benefits of the intervention other than adiposity? Is it cost-effective? Others constitute a bit of a "grab bag" research aims, valid enough in themselves, but without much of a common theme: the relationship between 24-h movement patterns and health outcomes, the eating environment. I would have liked to see an investigation into the mechanisms of the intervention: why has it been so successful when it did not change sleep duration?

The proposal is built on two new sleep paradigms: that sleep is part of a 24-hour day and involves trade-offs with other behaviours, and that characteristics of sleep other than duration (timing, variability, quality) may be important determinants of health.

Design and methods — Score: 5

The sample is quite large, and retention has been excellent. Because it is an existing sample, there are economies in not having to recruit participants.

This proposal will also marshall new measurement techniques, including multiple accelerometers. In principle, this is a good idea, but the lack of validation of these devices in this placement concerns me. While it shows promise for pattern recognition, at least in scripted behaviours, I would like stronger evidence that it is valid in free-living individuals. Still, as the researchers note, they can always fall back on the validated wrist-mounted device. The details about how the accelerometry data will be processed are very vague. I notice a study is underway, but it would give me greater confidence if has been completed.

The dietary experiment with the kids is clever.

The analytical procedures are excellent, particularly the use of compositional data analysis, a relatively new technique that Prof Taylor has pioneered. The researchers might also consider using CoDA fort body composition (which is obviously also a composition!) since they will have gold standard DXA measures.

I don't have much confidence in dietary measures in general, but the method they are using, a variant I think of LINZ24, is as good (or as bad) as any. Ioannides inter alios has pointed out some serious problems with nutritional epidemiology and its methods.

Wearable cameras in principle offer some possibilities, but to date they have been disappointing: enormous amounts of processing time, and automated processing has not been terribly successful. The collaboration with the "Spanish computer scientists" seems a bit pie-in-the-sky (or châteaux en Espagne).

Research impact Score: 6

The secondary studies offer great research potential: How do 24-h movements patterns relate to health outcomes? Can we develop pattern-recognition algorithms based on data from multiple accelerometers? Can AI be used to process images from wearable cameras? I'm more sanguine about some of these than others, but they are innovative and have very large potential methodological and knowledge implications.

In particular, the application of CoDA to 24-h movement data, particularly coupled with examination of multiple characteristics of sleep as covariates, is potentially paradigm-changing.

Team: research outcomes Score: 7

This intervention is striking because of the spectacularly large, and sustained, effect size despite a very light intervention. It will be fascinating to see if the effect is retained after 10 years. If it is, then it will be time for a large duplication study, with potentially major public health translational benefits.

The team is a well-established and internationally respected group, with a very impressive history of success in granting and funding. They have a long history of working together in closely-related areas. Each has an identifiable areas of expertise, and the skill set required is covered without omission or overlap. Where specific expertise is required (eg AI for image identification), it has been appropriately outsourced.

The research is of particular relevance to Maori because of the the high levels of overweight and obesity in that population.

General comments

Questions:

(1) Can the applicants provide evidence of the success of the 3-accelerometer system in identifying activities in free-living children?

(2) Can the applicants provide evidence of the success of AI in automatic identification of wearable camera images?

(3) What is the reliability and validity of the dietary recall methodology? Have the applicants considered DLW?

(4) Have the applicants considered using CoDA in treating body composition as a composition?

(5) What information will this study add to answer the burning question of WHY this intervention has succeeded so well?

Applicant peer review report

Health Research Council of New Zealand

Reviewer # 81

Proposal details

Title	Does a brief sleep intervention in infancy have long-term health benefits?
First named investigator	Professor Rachael Taylor (University of Otago)

Rationale for research Score: 6

The proposed project provides a highly unique and important opportunity to examine whether differences in BMI z-scores remain apparent 8-9 years after intervention - prior to puberty. The project also allows for the opportunity to answer a host of other important questions. This is important and justified research towards tackling childhood obesity. The project importantly follows-up a cohort of children (at 2 years of age) from a previous intervention study. Although an effect from the brief sleep intervention was apparent at 5 years of age, it is unclear as to why this was not seen at 3.5 years.

Design and methods Score: 6

The study design and methods are excellent. The initial RCT was a 'state-of the-art' intervention and design. The recruitment at follow-up at 3.5 and 5 years of age was excellent. It appears there will be ample study sample to conduct the follow-up primary analyses (BMI differences) proposed in this application.The methods to address the various secondary study objectives are very strong including valid measures and 'cutting edge' measurement protocols (eg AX3 accelerometers; wearable cameras).

Research impact Score: 6

There are relatively few obesity interventions with long-term outcomes for children. This study will provide important information on the potential benefits (eg obesity reduction) of child sleep interventions. If show effective in the long-term, the POI sleep intervention could be a cost effective strategy towards tackling the obesity problem in our population.

Team: research outcomes Score: 5

This is a very strong team who clearly have the diverse skills and track records to complete this research. Of note, the team has published 13 papers from the POI study.

General comments

Overall this is a very worthwhile project and well-written grant. The application became clearer to me after reading Taylor et al., (2017; Pediatrics) - reference 4. The initial RCT design and various analyses were quite complex.

Applicant peer review report

Health Research Council of New Zealand

Reviewer # 83

Proposal details

Title	Does a brief sleep intervention in infancy have long-term health benefits?
First named investigator	Professor Rachael Taylor (University of Otago)

Rationale for research **Score: 5**

This proposal is important as it highlights an innovative intervention to potentially combat obesity. There are two crucial points in the rationale that are cited with unpublished research. This reduces the impact of that evidence and drafts of that research should be made available for review.

Design and methods **Score: 5**

The design is comprehensive, but burdensome for the participant. The reimbursement is an appropriate one. No information is given on the validity or precision of the intake24. Although the time taken to review wearable camera photos will be likely reduced with the implementation of image recogntion software, no time estimation was stated.

Research impact **Score: 3**

There were credible pathways identified by the investigative team that may result in benefits for NZ.There is certainly scope to influence research abroad. However, policy influence and changes, although suggested by the teams presence and "proven expertise" on several guidelines groups were vague and gave no cited examples. The only conclusion to be drawn from this is that their influence has been negligible thus far.

There were hints of maximising the likelihood of impact beyond the productions of knowledge such as influencing research practices, however translation to health professional or public sleep practices is still some way off it seems as there was no evidence of this occurring.

Responsiveness to Māori placed the onus on others (externals) and not the investigative team. This section is asking how the proposal is responsive to Māori. There were plenty of passive statements e.g. "We hope that this body of work will ultimately lead to working with Māori", that insufficiently supported a responsive proposal. With no Māori investigator there is less chance of translation or research work with Māori. In these contemporary times, the throwaway statement of "seeking Māori postgraduates" is not an empowering strategy for Māori. To improve the Māori health research workforce, a Māori co-investigator that has a decision-making role on the investigative team needs to be directly supervising any Māori postgraduates taking part.

Team: research outcomes **Score: 4**

This team is impressive and has been able to deliver on their previous project outcomes. Their history of productivity is remarkable. Although impacts are perhaps overstated, the capability is certainly there. The networks and collaborations probably need to be improved in the health professional and early childhood realm if translation is to take place. Responsiveness to Māori is applicable in this case and the team does not appear to address this area.

General comments

no additional comments

Applicant peer review report

Health Research Council of New Zealand

Reviewer # 96

Proposal details

Title	Does a brief sleep intervention in infancy have long-term health benefits?
First named investigator	Professor Rachael Taylor (University of Otago)

Rationale for research Score: 7

This proposal is well written and well justified. The focus of the research proposal - prevention of childhood obesity has high

relevance and potential for impact globally and is a key public health issue in New Zealand where rates of childhood obesity and overweight are high, particularly among low income families and Maori and Pasifika peoples.

The POI study is internationally recognised as innovative and the findings of the effectiveness of a sleep intervention as preventative provides a range of positive directions in early intervention strategies and understanding of underlying mechanisms. The key aim of follow-up is important to demonstrate long-term effectiveness. Advances in measurement - particularly of 24 hour sleep and activity is significant.

Design and methods Score: 5

As a follow-up study the design is relatively simple. The key interest is in the new suite of measurement and advances in measurement. The measurement technology and battery of tests are comprehensive though I have some queries with regard to specification of measurement and analysis

1. Accelerometry and analysis of sleep - it was not clear to me how long measurement would be undertaken. Is this a day, a week or 2 weeks. The application focuses on 24-hour sleep, sedentary behaviour and activity but time-sampling is critical. Days of week can vary and more importantly regularity of sleep across days and weeks. Most important here is regularity of sleep patterns as irregularity, like jet lag, can affect hunger and satiety. Clarification is need on measurement duration and analysis of the sleep/activity data to ensure circadian mechanisms can be adequately captured

2. Screen time - I was concerned to ensure that measurement distinguishes between types - e.g. watching TV is necessarily sedentary while some screen time can be active and educative. How will this be analysed?

3. Mental Health- The suite of measures is comprehensive but I wondered if SDQ was the best measure given the data collections will be in clinics - a more extensive and psychometrically sound assessment would be possible though I understand use of SDQ gives comparability to many other studies

Research impact Score: 7

Childhood obesity, and attendant chronic disease in later life, is a critical public health issue for NZ and particularly for more socially disadvantaged groups and Maori and Pacific Island populations. The POI study has already made impact in this space and ongoing tracking is important to understand the potential of investment in interventions and to direct these investments to the right strategy.

There is a clear research impact pathway and a strong track record of influential publication from this team

Team: research outcomes Score: 7

The team is led by highly experienced researchers of international standing and makes provision for training of emerging leaders who have relevant skills and good outputs relative to career stage and opportunity.

Their combined track record is impressive and provides the surety of delivery of ongoing high quality research outputs and translation to policy and practice

General comments

The continuing follow-up of the POI study is important internationally. It promises to keep delivering innovative discovery in obesity prevention and management with implications for policy and practice in New Zealand but far beyond. The team is well able to deliver high quality work

I have some questions pertaining to measurement and analysis of data - particularly relating to analysis of circadian regularity. The study does not focus on exloratory mechanisms but my reading is that other attached studies are addressing these issues
